# My tooth is ill: about the mental representation of the concept of caries in children (phase I)

**DOI:** 10.1080/07853890.2021.1897365

**Published:** 2021-09-28

**Authors:** Maria do Rosário Dias, Maria Calejo Pires, Inês Nunes

**Affiliations:** ^a^Centro de Investigação Multidisciplinar em Psicologia da Saúde, Centro de Investigação Interdisciplinar Egas Moniz (CiiEM), Egas Moniz Cooperativa de Ensino Superior, Caparica, Portugal

## Abstract

**Introduction:**

Dental caries has a negative impact on children's oral health-related quality of life (QVRSO) [1], suggesting the need to understand how the oral clinical condition may interfere with the child's daily life. The acquisition of information that interacts with the child's mental schemas consolidates her ability to think inter and intrapsychic thought and knowledge of the world around her [2]. Same happens with the mental representation of the concept of caries that consequently influences the internalisation of salutogenic habits at the level of the biopsychosocial development of the infant patient [3]. In the present study we intend to evaluate the mental representation that children internalise about the concept of dental caries.

**Materials and methods:**

The sample of the present study consists of a total of 880 children, from 4 to 9 years, of both genders (51.7% girls, 48.3% boys). **Instruments:** (i) Sociodemographic questionnaire, data on age, sex, schooling and whether or not the participant has already resorted to a Dental Medicine consultation. **Procedures:** Data was collected at two moments: M1, where the child was asked to draw a *Healthy Tooth* on a sheet of paper and M2, where the child was asked to draw a *Sick Tooth* on another sheet using only a pencil of graphite and no rubber. Subsequently, the child was asked to respond to an open-ended questionnaire composed by three distinct questions, with the aim of evaluating the mental representation of the concept of (a) *Dental decay*, (b) *Healthy teeth* and (c) *Sick teeth*. NVivo software was used in order to carry out the content analysis of the written narrative, referring only to the first question (a) What is a dental decay for you?. The written answers were subjected to a content analysis grid that encompasses 13 elementary analytical categories.

**Results:**

Of note, 611 children who participated in the study had already attended a Dental Medicine consultation (69.4%), while 269 (30.6%) had never visited a health care unit. According to the results obtained in relation to the question (a) *What is a dental decay for you?,* 13 categories were created in which six were prominent, which seem to illustrate the mental representation of the concept of dental decay in this age group, namely: *Do not know* (18.5%), *Bugs/Monsters* (16.7%), *Teeth damage* (11%), *Bad oral hygiene* (9.6%), *Rotten tooth* (7.9%), *Teeth colouration* (7.3%), being curiously the category Bacteria, only referenced with a percentage of 2.2%.

**Discussion and conclusions:**

Since caries is an aetiological source of pain and malaise, it is important to recognise the mental perception of children about this concept, in order to contribute to the (re) conceptualisation of the concept of Oral Health Education, to the level of caries aetiology.
